# Virtual Touch tissue quantification cannot assess breast cancer lesions except for ductal carcinomas in situ and small invasive cancers: a retrospective study

**DOI:** 10.1186/s12957-015-0569-7

**Published:** 2015-04-15

**Authors:** Keiichiro Tada, Kotoe Nishioka, Yasuko Kikuchi, Takayoshi Niwa, Yasuyuki Seto

**Affiliations:** Department of Breast and Endocrine Surgery, The University of Tokyo Hospital, 7-3-1 Hongo, Bunkyo-ku, Tokyo 113-8655 Japan

**Keywords:** Breast neoplasm, Acoustic radiation force impulse, Virtual Touch tissue quantification, Ultrasonography

## Abstract

**Background:**

Virtual Touch tissue quantification (VTTQ) is a promising new technology that quantitatively determines the stiffness of tissue. However, the clinical impact of this device on the assessment of breast cancer is unclear.

**Methods:**

This study aimed to review the ultrasound records of patients with breast lesions where VTTQ was used to assess 123 normal breast tissues, 18 benign tumors, and 117 histopathologically confirmed breast cancers in a total of 129 patients. To determine the VTTQ value, a 5 × 5 mm region of interest was placed in the center of the area of interest, and the target lesion was measured at least three times by VTTQ.

**Results:**

Seventy-six percent of the malignant lesions could not be assessed using VTTQ. Among the malignant lesions, ductal carcinomas in situ (DCIS) and invasive breast cancers smaller than 1.6 cm tended to be ‘measurable.’ Only 17 and 1% of benign breast lesions and areas of normal breast tissue, respectively, were considered to be ‘unmeasurable’ (*P* < 0.001).

**Conclusions:**

A breast lesion that could not be quantitatively assessed by VTTQ was suspicious for malignancy. By contrast, DCIS lesions and small invasive breast cancers tended to be ‘measurable.’ These findings indicate that VTTQ may be a useful application for assessing breast tumors.

## Background

Ultrasonography is an essential diagnostic tool for breast neoplasms. B-mode ultrasound (US) imaging is a conventional, but especially important modality for clinical practice because of its high sensitivity [1]. However, B-mode imaging is limited because of its relatively low specificity [1]. Therefore, advanced US technologies, including Doppler ultrasonography [2] and contrast-enhanced ultrasonography [3], have been developed, and provide some diagnostic advantages.

Breast ultrasound elastography was introduced into clinical practice in order to support US diagnostic imaging. Manual compression of an area of interest using an US probe produces strain [4]. Assessment of the level of strain allows estimation of the hardness of a tissue, a characteristic that can be applied to the diagnosis of a breast lesion. A meta-analysis comparing the diagnostic performance of breast US elastography with B-mode imaging found that elastography had greater specificity [1]. However, manual compression must be performed properly, which requires training of the ultrasonographer. Furthermore, the stiffness of the lesion must be evaluated relative to the surrounding normal breast tissue [4].

A new type of elastography, Virtual Touch tissue quantification (VTTQ), which was recently introduced, quantifies the stiffness of breast tissue without using mechanical compression [5]. Acoustic radiation force impulse (ARFI) technology induces the mechanical excitation of tissue by means of localized impulsive radiation force, thereby propagating shear waves. The velocity of the shear wave is then calculated. The velocity of the propagated shear wave is related to the stiffness of the tissue. Shear wave speed generally increases with increasing tissue stiffness. Therefore, tissue stiffness can be easily quantified from the velocity of the shear wave.

Although this new technology appears to be a promising diagnostic modality, its clinical applicability to breast lesions is unclear. The finding of increased stiffness is thought to be associated with malignant breast tissue. However, previous studies have failed to confirm this hypothesis because VTTQ systems frequently failed to provide a stiffness value; in other words the VTTQ system displayed the reading ‘X.XX m/s.’ Therefore, it is important to identify the characteristics of breast lesions that allow VTTQ assessment.

In this study, we retrospectively investigated the VTTQ assessments of breast tissue, including normal tissue and benign and malignant lesions to determine which characteristics of breast tissue and breast lesions are associated with successful VTTQ assessment.

## Methods

### VTTQ measurement

**Figure 1:**
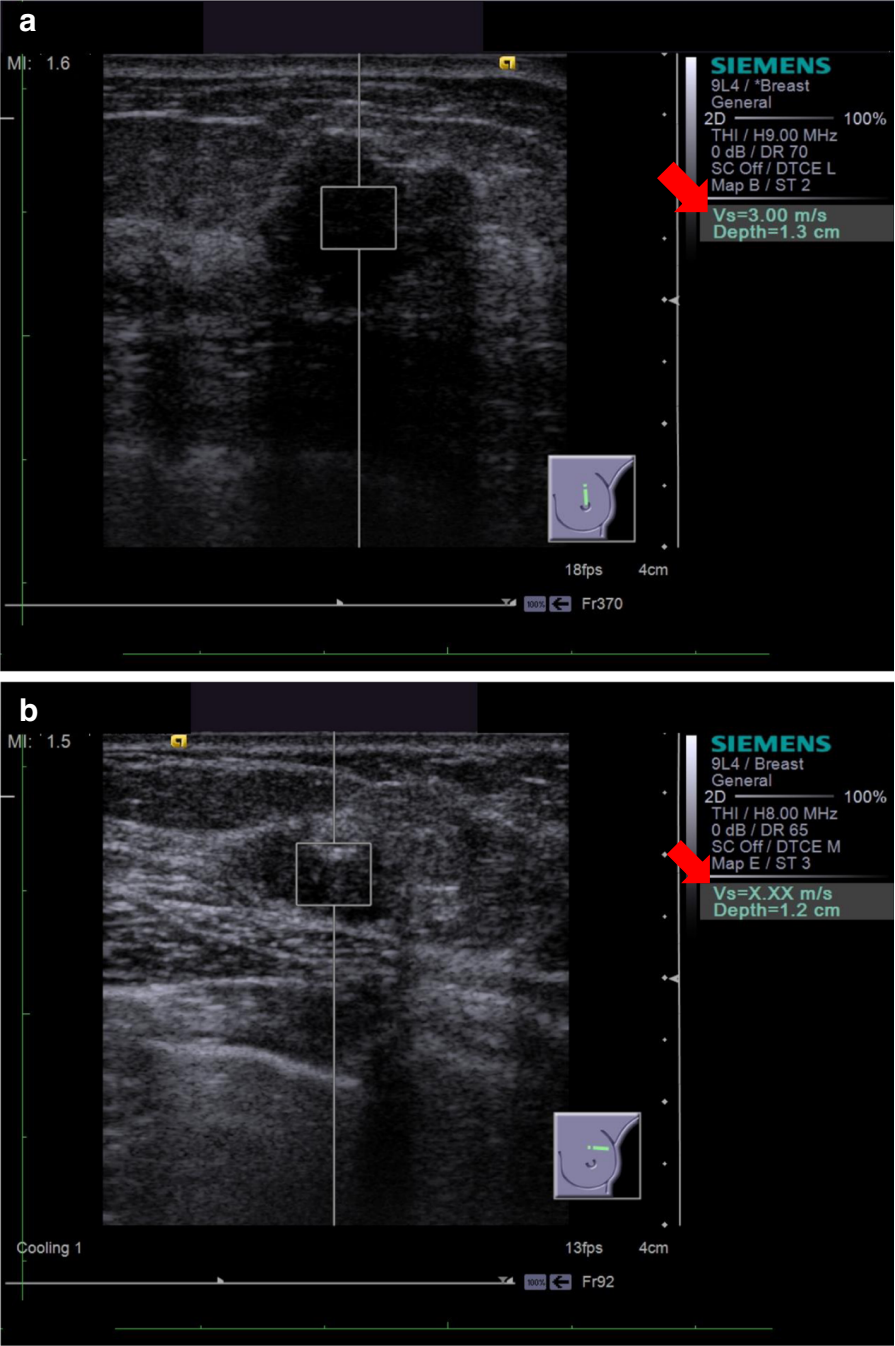
Typical Virtual Touch tissue quantification (VTTQ) images with results. During real-time ultrasonography, a 5 × 5-mm region of interest is placed on the area of interest. When VTTQ is activated, the display is frozen and the calculated VTTQ value is displayed **(1a)**. When VVTQ is unsuccessful, ‘X.XXm/s’ is displayed **(1b)**.

VTTQ was performed using the ACUSON S2000 ultrasound system equipped with the 9 L4 transducer (Siemens Medical Solutions, Mountain View, CA), according to a modification of the method published by Tozaki et al. [6]. Briefly, a 5 × 5 mm region of interest (ROI) was placed in the center of the area of interest (Figure 1a). The target lesion was measured at least three times. If every measurement was obtained successfully, the target lesion was classified as ‘measurable.’ The mean measurement was the final value for stiffness. If a measurement value was displayed as ‘X.XX m/s’ at least once, the lesion was classified as ‘unmeasurable’ (Figure 1b). Normal breast tissue adjacent to the lesion was tested in the same way. In a breast with several cancerous nodules, the largest and the second largest nodule were measured. Two operators (K.T. and K.N.), who each had more than 10 years of experience performing breast US imaging, performed these studies.

### Patients

According to our ultrasound registry, 1,033 patients underwent breast ultrasonography via the ACUSON S2000 ultrasound system from May 2011 to March 2013. Of these patients, 144 underwent VTTQ elastography. Of these patients, 112 were found to have 118 malignant lesions and 19 to have 19 benign lesions. Data from cases meeting the following criteria were extracted for analysis: 1) VTTQ assessment had been performed using the methods described in the previous section, 2) histopathologically confirmed breast cancer lesion, or 3) histopathologically or cytologically confirmed benign breast lesion. This study finally included 111 patients with 117 malignant lesions and 18 patients with 18 benign lesions. All the study patients were female. The mean ages of patients with breast cancer and benign lesions were 56.1 years (range 28 to 85 years) and 46.4 years (range 24 to 77 years), respectively.

### Statistical analysis

The chi-square test was used for categorical data. For numerical data, the Kruskal-Wallis test and Mann-Whitney test were used to compare multiple and two groups, respectively. Receiver operating characteristic (ROC) analysis was used to evaluate lesion size for measurability. *P* ≤ 0.05 was considered significant. IBM SPSS Statistics Desktop version 20 was used for analysis. Permission to perform this retrospective study was obtained from the ethical board of our institution.

## Results

A total of 135 areas of normal breast tissue in 129 patients were measured using VTTQ, and 123 areas among them were analyzed in this study. Twelve areas were excluded because they were tested less than three times. Furthermore, a total of 19 benign breast tumors were measured, and 18 of them were analyzed in this study. One benign mass was excluded because it was tested less than three times. There were 8 fibroadenomas, 2 cases of mastopathy, and 1 case each of inflammation, breast hamartoma, and breast cyst. For the remaining 5 cases, a fine needle aspiration biopsy was negative for malignancy. A total of 118 breast cancer lesions were measured. One mass was excluded because it was tested less than three times. The 117 malignant lesions that were analyzed included 13 cases of ductal carcinoma in situ (DCIS) and 104 cases of invasive carcinoma.

Table 1 shows the distribution of measurable and unmeasurable ROIs among the tested tissues of normal breast, benign breast tumors, and malignant lesions. Of the tested ROIs, 76% of malignant lesions, 17% of benign breast lesions, and 1% of normal breast tissues were unmeasureable (*P* < 0.001).

**Table 1:** **The proportion of lesions that were ‘unmeasurable’ according to normal, benign, and malignant regions of interest**

	**Unmeasurable**	**Measurable**	
Normal Breast Tissue	1 (1%)	122	123
Benign Lesion	3 (17%)	15	18
Malignant Lesion	89 (76%)	28	117
			*P* < 0.001

The percentages in parentheses indicate the proportion of ‘unmeasurable’ normal, benign, and malignant lesions. Chi-square test was used for statistical analysis.

Table 2 shows the frequencies of unmeasurable DCIS and invasive breast cancer lesions. More cases of invasive breast cancers were unmeasurable than cases of DCIS (*P* = 0.002).

**Table 2:** **The proportion of ‘unmeasurable’ regions of interest that were invasive cancer or DCIS lesions**

	**Unmeasurable**	**Measurable**	
Invasive cancer	84 (81%)	20	104
DCIS	5 (39%)	8	13
			*P* = 0.002

The percentages in parentheses indicate the proportion of ‘unmeasurable’ invasive cancer or DCIS lesions. Chi-square test was used for statistical analysis.

DCIS: ductal carcinoma in situ.

**Figure 2:**
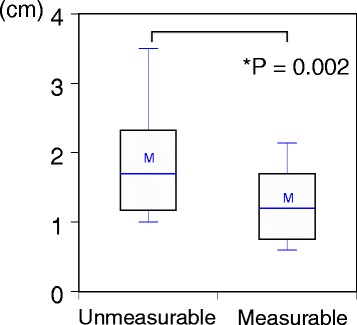
Box-and-whisker plots of breast lesion sizes according to whether lesions were measurable or unmeasurable by Virtual Touch tissue quantification. Nonparametric analysis found a significant difference in the sizes of measureable versus unmeasureable lesions. The horizontal line in the box indicates the median size of the samples. The upper and lower sides of the box represent the 75th and 25th percentiles, respectively. The upper and lower sides of the whiskers represent the 90th and 10th percentiles, respectively. ‘M’ is the mean value of the samples.

**Figure 3:**
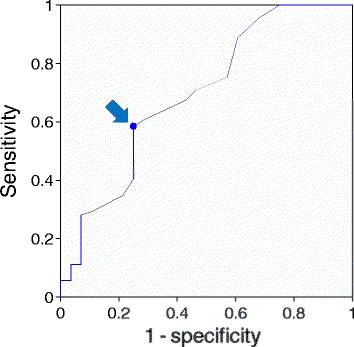
Receiver operating characteristic curve of breast lesion sizes according to whether lesions were measurable or unmeasurable by Virtual Touch tissue quantification. The arrow indicates the Youden index.

Figure 2 shows the relationship between tumor size and measurability for lesions of invasive breast cancer. Smaller lesions of invasive breast cancer were more likely to be measurable (Mann-Whitney test, *P* = 0.002). Figure 3 shows the ROC curve of lesion size and measurability. The Youden index was 1.6 cm, which was the estimate for the cutoff value for the size of tumors assessable using VTTQ.

**Figure 4:**
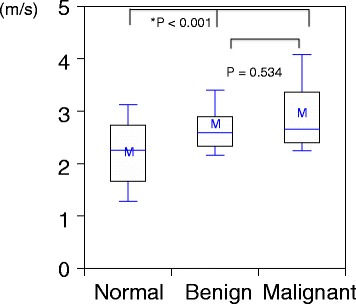
Box-and-whisker plot of Virtual Touch tissue quantification of the region of interest in normal breast, benign breast lesions, and malignant lesions. There was a significant difference between the velocity of the shear waves produced by these three types of lesions according to nonparametric analysis. However, there was no significant difference between benign and malignant lesions. The horizontal line in the box indicates the median of the samples. The upper and lower sides of the box represent the 75th and 25th percentiles, respectively. The upper and lower sides of the whiskers represent the 90th and 10th percentiles, respectively. ‘M’ is the mean value of these samples.

Figure 4 shows the distribution of mean VTTQ values of measureable normal breast tissues, benign lesions, and malignant lesions. The degree of elasticity decreased from normal to benign to malignant lesions. Nonparametric analysis found a significant difference between the three types of tissues measured (*P* < 0.001). However, the difference between benign and malignant lesions was not significant (*P* = 0.534).

## Discussion

We investigated the impact of VTTQ on breast imaging, demonstrating that this new technology has some value.

An advantage of our study is that our measurement methodology was easy to perform. That is to say, the ROI was placed in the center of the area of interest, three measurements were taken, the value for stiffness was the mean of three successful measurements, and lesions shown as ‘X.XX m/s’ at least once were considered to be unmeasurable. The value for shear wave velocity is often displayed as ‘X.XX m/s,’ and that occurs if the tissue in the ROI is heterogeneous, the amplitude of the shear wave is low, the noise-to-signal ratio is high, or the velocity of the shear wave is extremely high [7,8]. Suggestions for managing ‘X.XX m/s’ readings vary and include the following: perform repeated measurements (up to 23) [9], place the ROI on the margin of the area of interest [6], and substitute 9.10 m/s for ‘X.XX m/s’ [10,11]. The authors of these studies paid particular attention to quantity and were reluctant to accept measurement failure. Because we accepted the possibility of measurement failure for malignant lesions, we used a simple protocol that we believe will be easy to reproduce at every institution.

Our results indicate that a breast lesion that could not be quantitatively assessed by VTTQ was suspicious for malignancy. Seventy-six percent of malignant lesions were characterized as unmeasurable, which was a significantly higher rate than seen for benign lesions or normal tissues (*P* < 0.001). Furthermore, the high rate was similar to rates observed by other studies [6,10]. Although an unmeasurable lesion cannot definitively be determined to be benign or malignant, our finding is very useful for clinical practice.

Another advantage of our study was that the small invasive breast cancers and lesions of DCIS were associated with successful VTTQ measurement. We can reveal these findings because our investigation included more than 100 cases of breast cancer. These findings are important for successful VTTQ measurements of breast lesions less than 1.6 cm or DCIS lesions.

The VTTQ technology has several advantages for breast imaging. First, this technology is easy to use and noninvasive. Second, the measuring device does not require mechanical compression, so that training is not needed for ultrasonographers. Last, this type of elastography can produce absolute values for stiffness. These features make this technology highly advantageous compared to the current breast imaging systems.

However, for the accurate evaluation of breast tumors by VTTQ elastography, additional studies of patients with various breast lesions, including mucinous tumors, necrotic tumors, intracystic tumors, and microcalcification-containing lesions are needed. The technology used for measurement also needs improvement. As mentioned previously, ‘X.XX m/s’ is displayed for a variety of reasons [6,9-11]. The reasons why malignant lesions are unmeasurable must be clarified for clinicians. Furthermore, our study found that there was no significant difference in the degree of stiffness between the measureable benign and measureable malignant lesions. Although we believe that the small number of measureable cases could have accounted for our result, further investigation is needed to resolve this issue.

## Conclusions

A breast lesion that could not be quantitatively assessed by VTTQ was suspicious for malignancy. By contrast, lesions of DCIS and small invasive breast cancers tended to be ‘measurable.’ These findings indicate that VTTQ may be a useful application for assessing breast tumors.

## Abbreviations

DCIS: ductal carcinoma in situ
ROC curve: receiver operating characteristic curve
US: ultrasound
VTTQ: Virtual Touch tissue quantification
